# Non-Rigid Registration for High-Resolution Retinal Imaging

**DOI:** 10.3390/diagnostics13132285

**Published:** 2023-07-06

**Authors:** Mircea Mujat, James D. Akula, Anne B. Fulton, R. Daniel Ferguson, Nicusor Iftimia

**Affiliations:** 1 Physical Sciences, Inc., 20 New England Business Center, Andover, MA 01810, USA; dan@fergusonrd.com (R.D.F.); iftimia@psicorp.com (N.I.); 2 Department of Ophthalmology, Boston Children’s Hospital, Boston, MA 02115, USA; james.akula@childrens.harvard.edu (J.D.A.); anne.fulton@childrens.harvard.edu (A.B.F.); 3 Department of Ophthalmology, Harvard Medical School, Boston, MA 02115, USA

**Keywords:** adaptive optics, retinal disease, scanning laser ophthalmoscopy

## Abstract

Adaptive optics provides improved resolution in ophthalmic imaging when retinal microstructures need to be identified, counted, and mapped. In general, multiple images are averaged to improve the signal-to-noise ratio or analyzed for temporal dynamics. Image registration by cross-correlation is straightforward for small patches; however, larger images require more sophisticated registration techniques. Strip-based registration has been used successfully for photoreceptor mosaic alignment in small patches; however, if the deformations along strips are not simple displacements, averaging can degrade the final image. We have applied a non-rigid registration technique that improves the quality of processed images for mapping cones over large image patches. In this approach, correction of local deformations compensates for local image stretching, compressing, bending, and twisting due to a number of causes. The main result of this procedure is improved definition of retinal microstructures that can be better identified and segmented. Derived metrics such as cone density, wall-to-lumen ratio, and quantification of structural modification of blood vessel walls have diagnostic value in many retinal diseases, including diabetic retinopathy and age-related macular degeneration, and their improved evaluations may facilitate early diagnostics of retinal diseases.

## 1. Introduction

Adaptive optics (AO) has been applied to ophthalmologic imaging to enhance the resolution for in vivo imaging at the cellular level [1,2,3,4]. The building blocks of retinal microstructures such as cone and rod photoreceptors, retinal pigment epithelial (RPE) cells, blood cells, and microvasculature need to be accurately identified, counted, segmented, and mapped for diagnostic purposes. In general, multiple images are acquired at the same location and are registered. The registered image stacks can then be averaged to improve the signal-to-noise (SNR) ratio or analyzed to reveal temporal dynamics. For small patches of the order of half a degree, image registration is relatively easy using simple cross-correlations. However, as the image size increases to 1–2° in flying-spot scanning laser ophthalmoscopy (SLO) [5,6] and even more to 3.5 × 5°, as in line-scanning retinal imagers [7], more sophisticated image registration techniques are needed to remove image distortions and motion artifacts. Hardware image stabilization [8,9,10,11,12,13] has been developed and proved to be sufficiently good that simple software routines that could align and blend the recorded images. Concomitantly, a software version, strip-based registration [14,15,16] has been developed, based on the assumption that deformations along the strip are relatively negligible due to the high speed of scanning with resonant scanners while torsional motion is neglected altogether. Yet, the living eye is not a rigid sphere, and imaged fields are neither flat nor insensitive to changing optical/geometrical perspectives that accompany motion; rather, the eye is a fluid-filled organ subjected to physical (and thus optical) distortions, as it is periodically pumped with blood and intermittently pulled by muscles. It is reasonable to expect microns of deformations in all directions over 0.5–1.5 mm field sizes, not only orthogonal to or along the strip. If the deformation from one end of the strip to the other is comparable to the cone size, strip-based registration will blur out the cones instead of providing the hoped for improvement in image analysis [17]. To correct for similar types of local deformations, non-rigid registration methods have been successfully used in medical imaging [18,19,20,21,22,23]. In retinal imaging in particular, non-rigid registration techniques have been used to improve visualization of retinal capillaries using speckle-variance optical coherence tomography (OCTA) [24] or AO scanning laser ophthalmoscopy [25]. We propose here the use of a non-rigid registration method to register stacks of high-resolution retinal images to improve the quality of the final average image. Our procedure illustrated here on cone images can be applied for images of other retinal microstructures as well.

## 2. Materials and Methods

### 2.1. The Imaging System and Procedure

The multimodal AO retinal imager (MAORI) used here has been described previously [5,13]. It included both AO-corrected SLO and optical coherence tomography (OCT) imaging channels that shared a common galvanometer for vertical (slow) scanning and acquiring perfectly registered images simultaneously. MAORI uses broadband (40–60 nm) superluminescent diodes (SLD) for SLO (at 760 nm) and OCT (at 850 nm). It has been designed for a 7.5 mm beam diameter at the eye pupil that provides ~2.5 µm lateral resolution in a normal human eye in both AO-SLO and AO-OCT images. The SLO images (1000 × 1024 pixels) discussed in this paper were acquired at 28 Hz frame rate. Square SLO images with a size adjustable from 0.5 to 3 degrees (in high-resolution mode) can be positioned anywhere within the field of view of the instrument (fixation target) covering ~33 degrees. Additional features of MAORI include an auxiliary LSO imager for global orientation, two pupil configurations for a variety of subject species (including humans, large primates, and small animals), pupil camera, high-speed GPU processing of OCT and WS images, and a LCD-based fixation target. MAORI is a powerful research and clinical platform for high resolution imaging of cells and fine structure in the retina.

### 2.2. Human Subjects and Imaging Procedure

The multimodal AO retinal imager was tested at Boston Children’s Hospital. A human subject protocol was approved by Boston Children’s Hospital IRB prior to imaging, and all subjects gave informed consent to be imaged. Some of the subjects with small pupils were dilated to enhance the AO correction. The imaging sessions followed a set protocol, including several scans with varied sizes and at different eccentricities. The data analyzed in this paper were obtained from three normal volunteers, A—23-year-old female; B—38-year-old male; and C—19-year-old female, and are shown here for illustration purposes only.

### 2.3. Deformations

Images were acquired at 28 frames per second. The fast scan for the SLO images was generated with a resonant scanner (14.5 kHz). Either a nonlinear pixel clock or post-processing in software [26,27,28] was needed to properly dewarp the image. Software dewarping assumed that the resonant scanner followed a sinusoidal law of motion that was properly calibrated and was well known with respect to the image. Any error here would result in varying pixel spacing occurring laterally across the image, and when a certain structure of the retina showed up in different parts of different frames it looked like it changed size. In addition, motion artifacts responsible for image deformations included fast eye saccades that generated intra-frame distortions. Electronic noise and optical distortions also affected the image. Jitter in the line trigger when acquiring the SLO image and noise or non-linearity in the slow scan in addition to errors in dewarping generated small local distortions that needed to be eliminated when averaging multiple frames that were acquired tens of milliseconds to several seconds apart. Simple, rigid overlap would add blur to the average image.

**Figure 1:**
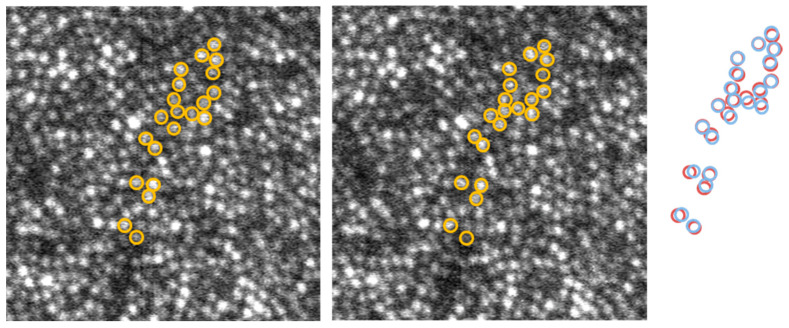
Two consecutive frames in an image stack (See Video S1) with a few cones encircled. The two cone patterns are overlapped on the right side (left image—blue, right image—red), illustrating different displacements. Volunteer A. Image size—120 µm; eccentricity—0.16 mm nasal.

Blood flow in capillaries above the photoreceptor layer distorted the image of the cones. In general, capillaries filled with red blood cells cast shadows that hide cones underneath, whereas, occasionally, white blood cells act as moving magnifying glasses, causing the cones underneath to become visible. These events also create local distortions wherein the apparent distance between cones on opposite sides of the capillary changes; the reverse effect, where cones are mostly visible, and a moving shadow transiently hides cones, is also occasionally observed. Figure 1 shows two consecutive frames from an image stack distorted differently by a blood cell flowing through a capillary. The right side of Figure 1 shows the overlap of the two patterns of cones circled in the two frames. Clear motion in different directions can be observed; this is also illustrated in frames 3–5 of Video S1.

### 2.4. Preliminary Steps of Image Processing

**Figure 2:**
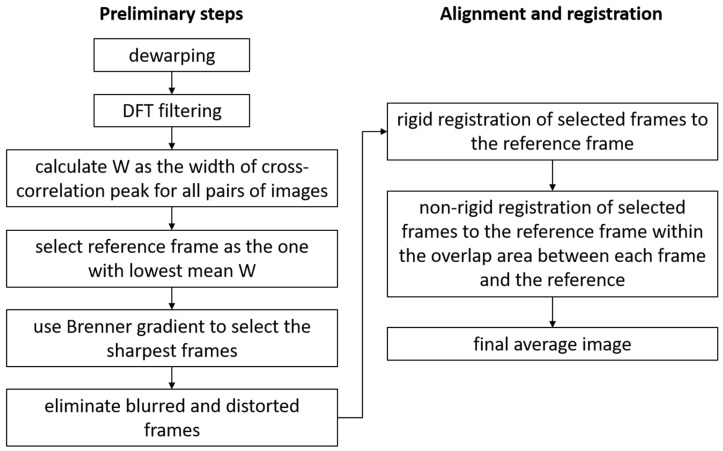
Flowchart illustrating all the steps in image processing.

Assuming that the dewarping was performed either in hardware or as a pre-processing software step, the first step in post-processing was to analyze the images to automatically select the best images and to eliminate the most distorted frames (due to blinks, saccades, intra-frame eye motion). All images were filtered to improve their contrasts and remove background intensity variations. A Discrete Fourier Transform (DFT) filtering procedure was used, in which a disk mask was applied to keep the ring associated with the photoreceptors. The DC was removed as well by filtering out the center peak of the DFT. All the steps of image processing are illustrated in the Figure 2 flowchart.

The next step was to find the best frame in the data set, a reference frame, which would serve as a template to which all the other frames would be aligned to. This was performed based mostly on the width of the cross-correlations of pairs of images. Similar images typically had high and sharp cross-correlation peaks, whereas dissimilar images had low and broad cross-correlation peaks. A cross-correlation of 2D images was a 2D image. The peak of the cross-correlation indicated the best overlap of the two images and had a more or less conical shape. In that sense, we calculated W as the diameter of the circular section through the peak going down from the apex (maximum) to 90% of the apex height. The peak location provided the x/y shifts of one image with respect to the other one. W used here as a measure of the sharpness of the cross-correlation peak was relatively insensitive to intensity variations between frames and was mostly related to the similarities of the structures contained in the overlapping regions of the two frames. W was calculated for all pair combinations in an image stack, and the mean value was obtained for each image, given all correlations between that specific image and all the other images in the set. The image with the lowest mean was selected as the reference frame since it had the best similarity with the most frames in the dataset.

Another problem that affected the image quality was the focus variations across a dataset. As AO is a dynamic process, and the eye accommodation might not always be paralyzed during imaging, the image sharpness varies among images in a dataset acquired in a clinic. Automatic selection of the sharpest images to be averaged was desirable, and, certainly, the template for alignment should be among the sharpest images in the stack. Brenner gradient (BG) [29,30] was used here as a figure of merit to select the sharpest images and to ensure that the reference frame selected based on W is among the sharpest images in the set. The threshold (*thr_w*) for W was set as the mean plus one standard deviation (*std_w*) for the W values obtained by cross-correlating the reference frame with the entire stack, and the frames with W > *thr_w* were considered distorted. Additionally, frames with BGs smaller than 75% of the reference frame’s BG were considered too blurred. The combination of distorted and blurred constituted the invalid frames to be eliminated from the alignment and average process.

The purpose up to here was to eliminate the most distorted frames and Keep the most similar Images to be aligned and averaged. One caveat was that the threshold criteria described above would sometimes eliminate more images than needed. For example, for a stable eye with few motion artifacts, most images were quite similar, and the standard deviations were quite narrow. Therefore, images with small distortions would be eliminated since their metrics fell less than one *std* away from the mean of the set. We preferred to err on the side of eliminating more images than needed to ensure that the algorithm worked unassisted on a large range of real patient data and not only on the best fixators. In any event, the best quality image sequences needed relatively few images to produce high-quality averages.

### 2.5. Non-Rigid Registration

**Figure 3:**
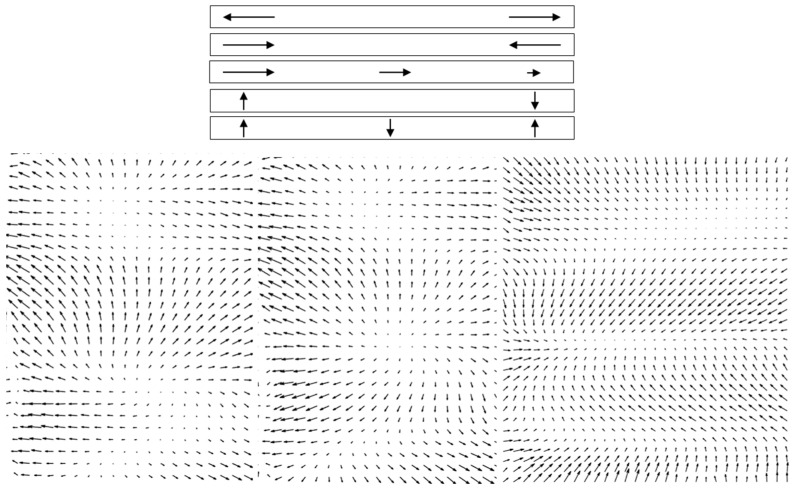
(**Top**)—examples of possible deformations of strips (top-to-bottom): stretching, compression, gradient, rotation, and bending; (**bottom**)—examples of local displacement for non-rigid registration of pairs of frames clearly illustrating individual deformation types.

The images in a stack that needed to be registered were generally acquired several tens of milliseconds to several seconds apart. As intra-frame distortions were evident, it was reasonable to expect inter-frame distortions as well. Strip-based registration could not account for deformations of the type illustrated schematically in the top of Figure 3. Stretch, contraction, or gradient along the strip; rotation and bend of the strip; and combinations of these deformations could occur. To compensate for these local deformations, we applied a non-rigid image registration technique [31] that improved the quality of the processed images for mapping cones. By using local grid deformations, non-rigid registration accounted for stretching and compressing in different directions in different parts of the image from frame to frame. The method was based on polynomial expansion to approximate the local signal, in which the coefficients were obtained through a weighted least square fit performed iteratively. The deformation field was accumulated at each iteration, and an example is shown in Video S2. The difference between the reference frame and a new image is shown in the right panel of Video S2, illustrating the cone displacement as black and white patterns, like shadows whose orientations indicated the directions of relative motion. Perfect overlap of a pixel between frames shows as gray. As the new image is subtracted from the reference frame, the black spots illustrate the positions of cones in the new image moving towards the white spots that illustrate the positions of the cones in the reference frame. The accumulated displacements over all iterations for three pairs of images are illustrated as quiver/velocity plots (MATLAB) in the bottom panel of Figure 3. These examples clearly demonstrate the need for local correction of the deformations that occur between a reference image and the subsequent images.

**Figure 4:**
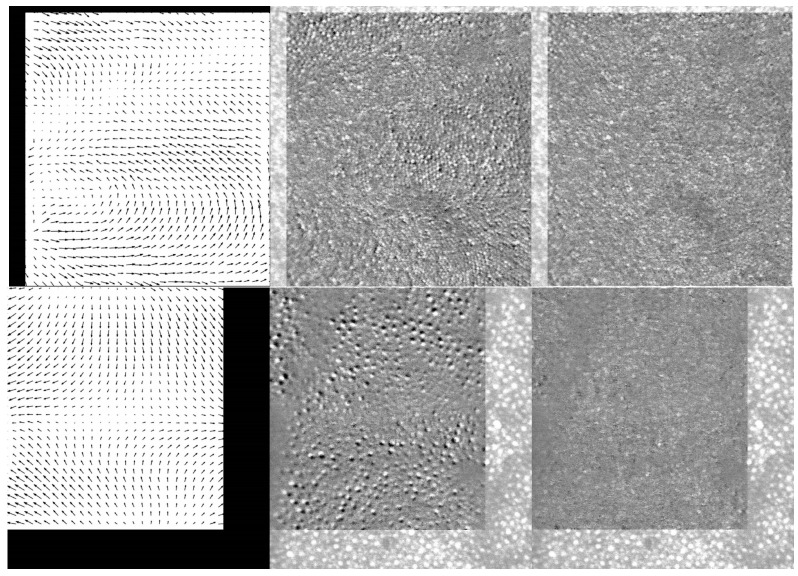
Two examples of the motion field (**left**), difference image between two registered frames using rigid (**center**) and non-rigid (**right**) registration (See Videos S3 and S4). Volunteer B.

Figure 4 shows two examples of the deformation field (left) and the difference image between the reference frame and the new frame for rigid (center—DR) vs. non-rigid (right—DNR) registration. The associated media (Videos S3 and S4) illustrated a large range of local deformations, some of which could have been corrected with a strip-based registration algorithm. However, many frames included deformation types (as illustrated in Figure 3) that required non-rigid registration. The center image (difference between the reference frame and a new image) shows cone displacements in different directions, whereas the cones are better aligned, and the difference image is much more uniform, in the right image (difference between the reference frame and non-rigid deformed new image). For the rigid registration (center), simple 2D cross-correlation was used to determine the lateral shift between the images, whereas the non-rigid registration was applied in the overlap region following the rigid alignment of the new image. In both examples in Figure 4, these images illustrate the improvement in uniformity across the whole frame provided by non-rigid registration.

The non-rigid registration might fail in some parts of the image. Sometimes, the DR image shows a black spot (new frame) in between two white spots (reference frame), mainly at the edge of the overlap region. The algorithm gets confused as to which direction to move the black spot and fails to properly register the new frame. In addition, the registration fails if the local displacement is too large. In these regions where registration fails, the image difference (DNR) has large values, whereas the image difference has small values in regions where the registration is good. These differences can be estimated either with a local standard deviation filter or a simple rectification filter defined as a local mean of the absolute value of the difference image (A = *abs*(DNR)). The second option is significantly faster in MATLAB than the first one. One can set a threshold for how good the registration is as *mean*(*A*) *+ std*(*A*) and filter out the regions that do not register well (the examples in Figure 4 are shown without such filtering). The expectation is that each region of the reference frame will have enough frames that will average well to provide the image quality improvement.

## 3. Results

**Figure 5:**
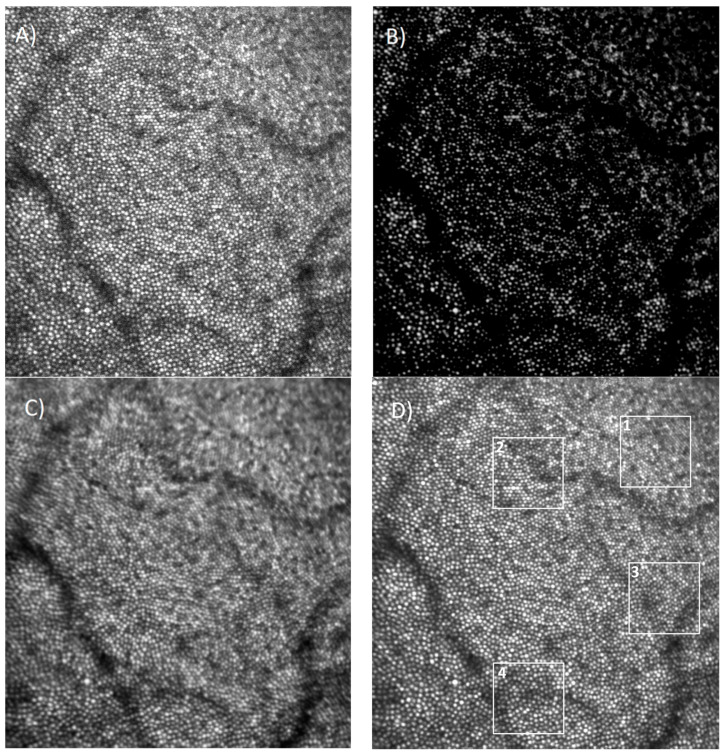
Reference frame (**A**), filtered image (**B**), and average image for a stack of 32 frames (out of 64) using rigid registration (**C**) and non-rigid registration (**D**). Volunteer C. Image size—442 µm, eccentricity—0.22 mm nasal/inferior. Regions 1 to 4 shown magnified in Figure 6.

Figure 5 shows the result of non-rigid registration of 32 frames selected from a stack of 64 frames, as described above, capturing a 1.5 × 1.5° field. The image with the best overall W in the stack was selected as the reference frame (Figure 5A). All other images were registered to this frame. Figure 5B shows the filtered reference frame. Figure 5C is the average image obtained using rigid registration only, and Figure 5D is the average image after non-rigid registration. Compared to the reference frame, rigid registration produces a blurred image, whereas non-rigid registration generates an image with significantly improved cone definition.

**Figure 6:**
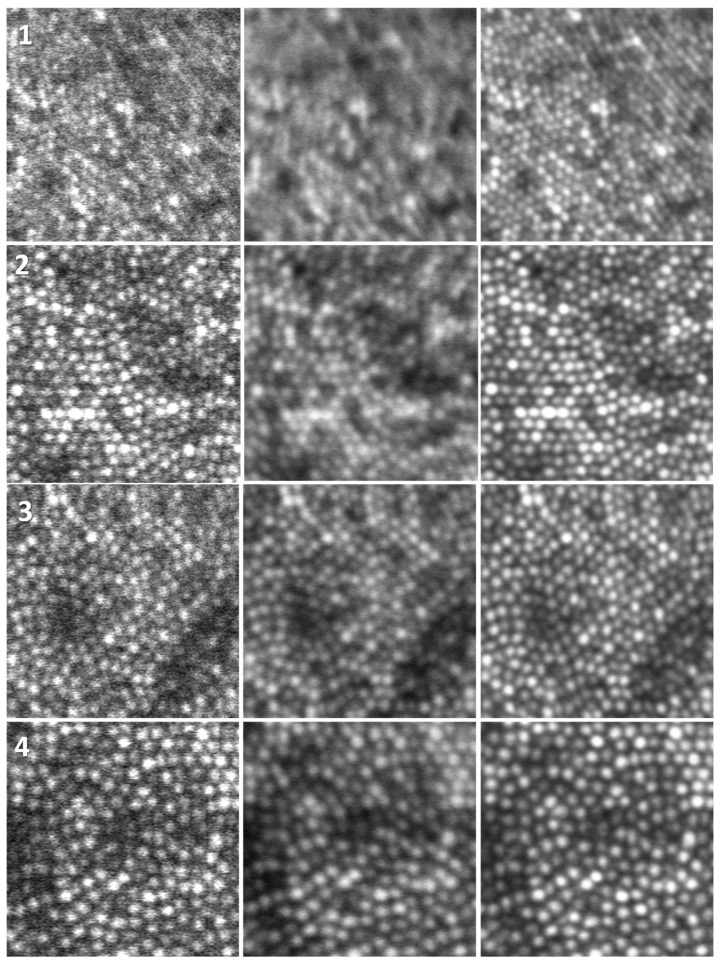
Reference frame (**left**), average image using rigid registration (**center**), and non-rigid registration (**right**) for the four regions delineated in Figure 5 (1 to 4 from top to bottom). Image size—88 µm. Eccentricity 0.12 mm, 0.27 mm, 0.29 mm, and 0.49 mm for 1 to 4, respectively.

Figure 6 shows a magnified version of the four areas delineated in Figure 5 by the four white squares. The center column of Figure 6 shows the rigid registered average, the right column is the non-rigid registered average, whereas the left column shows the corresponding area in the reference frame (areas 1 to 4 shown top to bottom). Cones that are not visible in the reference frame are nicely defined and can be easily counted and mapped in the non-rigid average image.

The cone mosaic is much more distinguishable; the cones are rounder, easier to identify and count, and even foveal cones become identifiable. A cone-counting algorithm has a much better chance to identify and count these cones compared to analyzing individual images. However, it should be noted here that non-rigid registration does not improve errors in the AO corrections. Dewarping artifacts are still visible in the left column of Figure 6.

**Figure 7:**
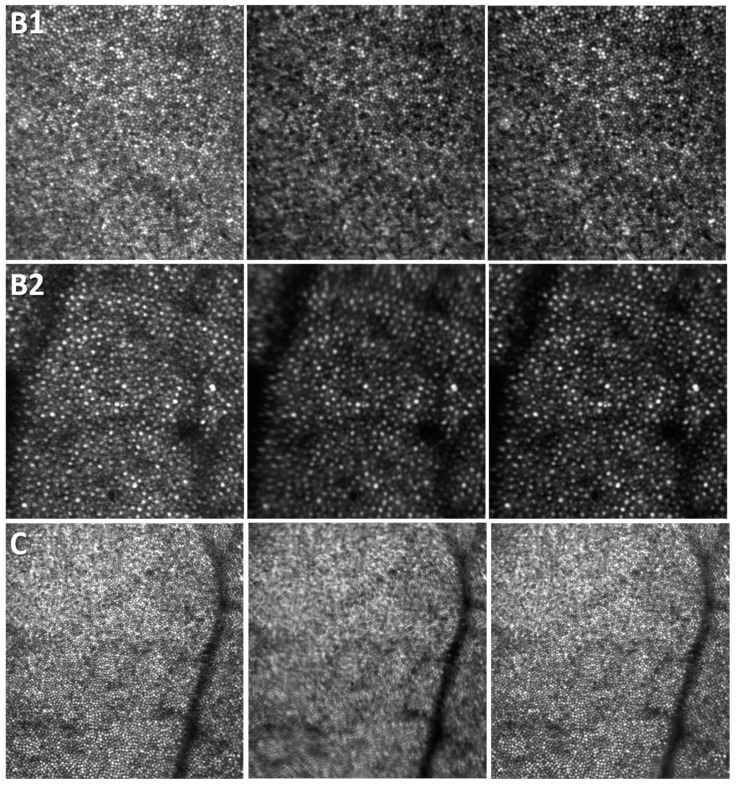
Examples of the reference frame (**left** column), rigid (**center** column), and non-rigid (**right** column) registration average. Volunteer B (B1 **top**, B2 **center**) and C (**bottom**). Image size: B1 and B2—293 µm, C—442 µm. Eccentricity: B1—0.15 mm temporal/superior, B2—1.4 mm temporal/superior, C—0.22 mm temporal/inferior.

The averaging results for the data shown in Figure 4 are illustrated in Figure 7. The top line (B1) in Figure 7 corresponds to the top data in Figure 4, whereas the center line (B2) in Figure 7 illustrates the results for the bottom data in Figure 4. The bottom line (C) in Figure 7 is from a different scan for the same volunteer analyzed in Figure 5 and Figure 6. The left column in Figure 7 shows the reference frame, the center column is the rigid registration average, and the right column is the non-rigid registration average image. The definition of the cones is clearly improved, and cones that are not visible in the reference frame become visible and nicely defined in the final non-rigid registration average image. The rigid registration average exhibits significant blurring. Clearly, different scans are affected differently by motion artifacts, and the final rigid registration average exhibits different levels of blurriness. Subjectively, in the center column of Figure 7, the top image shows less degradation as compared to the center image, whereas the bottom image is definitely unusable. However, in all cases, non-rigid registration undoubtedly provides clear improvement in cone definition and mosaic illustration.

## 4. Discussion

Stable eyes with relatively few small distortions in the acquired images are ideal; however, the reality in the clinic is different. Real data exhibit blinks, saccades, drifts, losses of fixation, focus changes, intensity variations among or within frames, or combinations of these effects. Repeating the scan is not always an option and does not always solve the problem; therefore, attempts need to be made to extract useful information from the available data. One of the most challenging tasks is to automatically identify and remove distorted frames.

There are many reasons for the complex deformations discussed here. A spectrum of eye motions from saccades to drifts and fixation losses contribute to both intra-frame and inter-frame distortions. There are also opto-mechanical influences due to the non-rigid construction of the eye, periodically pumped with blood in pressurized vessels and pulled by a set of powerful muscles that can induce dynamics in the globe and local distortions in the photoreceptor layer. Electronic noise, trigger noise, and galvanometer instabilities add local deformations in the recorded images. The images of the cone mosaic become distorted and need to be corrected to achieve optimal registration. The algorithm described here was designed to work automatically and unassisted on a large set of patient data. Distorted frames are automatically removed from the data set, using parameters (thresholds) calculated based on the dataset using means and standard deviations. A reference frame is selected automatically based on its similarity to the other frames and the image’s sharpness; all other frames are registered to it. Rigid registration through cross-correlation provides the initial alignment of the main features. Non-rigid registration then corrects the local deformations of the images to provide proper registration of the microscopic features like cone photoreceptors or capillaries. The final blending of the registered stack of images shows clear improvement over rigid registration, also correcting for a large range of distortions not accounted for in strip-based registration algorithms. Cones not visible in individual images or blurred with rigid registration become clearly visible with non-rigid registration and have a better chance to be counted and mapped by automatic cone-counting algorithms.

The process runs unsupervised on any number of scans acquired in the clinic on a wide range of healthy or diseased eyes. Some of the thresholds (the 90% peak height for W, or 75% for BG) were set empirically based on the algorithm’s performance on a large number of clinically relevant scans, whereas other thresholds were set based on means and standard deviations within the processed stack’s images. The automatic selection of the most similar images, elimination of the most distorted ones, as well as automatic selection of the reference frame enable efficient processing of multiple scans for a complete analysis of an imaging session. An additional routine has been developed that allows an expert grader to manually select the desired frames. This feature is valuable if the subject switched fixation between two relatively stable fixation positions during the acquisition of an image stack, and the algorithm selected one reference frame for the location with the most similar frames. The other location might also have valuable retinal information, and the grader has the option to manually select and average those frames.

The images shown here illustrated non-rigid registration improvements in photoreceptor imaging. Equally important, the method has been applied to offset aperture/split detection images [32] that visualized capillary networks and the cellular details of blood vessel walls. Improved definition of cellular details provided by non-rigid registration enabled segmentation of these structures and quantification of characteristic geometrical parameters. The lumen diameters and the wall thicknesses of blood vessels could be evaluated, and local malformations such as thinning or thickening of the vessel wall could be measured and monitored longitudinally. Local wall thinning could be an indication of potential wall rupture with damaging effects on vision. The wall-to-lumen ratio has diagnostic value for diseases that have vascular consequences and has been investigated for a long time in retinal imaging, however, without the cellular resolution afforded by AO.

The automatic co-registration of the SLO and OCT images provides an additional advantage for image registration: the two images are orthogonal to each other and, therefore, 3D registration could be possible for the SLO and OCT images, a feature that is missing in AO-SLO- or AO-OCT-only imagers. To obtain 3D OCT rasters, the image pair SLO/OCT was scanned laterally (perpendicular to the B-scan), and there was significant overlap between consecutive SLO images. Alignment of the SLO images could the necessary correction for proper en face placement of the OCT images, whereas the axial alignment of the OCT images could offer the correction for axial motion that could be used for the analysis of the SLO stack. While the SLO alignment was not used here for OCT correction, and the OCT information was not used for SLO alignment in this paper, the simultaneous acquisition of the OCT and SLO images represents a unique feature of MAORI that may enable cross-modal 3D registration.

## 5. Conclusions

Simple cross-correlation provides the necessary shift for rigid registration of high-resolution retinal images. However, that is not sufficient for averaging the images shown here. Images were aligned and averaged using rigid registration, and the result was a blurred image. In the past, we have used strip-based registration [7] to improve the final average image, as compared to simple full-frame rigid registration. However, we soon came to realize that strip-based registration did not work satisfactorily over a wide range of real patient data for the reasons explained in this paper. Therefore, a new type of correction for high-resolution retinal images based on non-rigid registration that accounted for local distortions in the recorded images was introduced here. Distortions included local image stretching, compressing, bending, and twisting due to a number of causes. The final average image showed significant improvement in the definition of cone photoreceptors, as compared to individual images and rigid registration performed by simple cross-correlation.

The non-rigid image registration procedure described here provided improved definition of retinal microstructures that were visible in the high-resolution retinal images, such as cones, rods, capillary networks, aneurysms, microglia, macrophages, hyalocytes, mural cells, endothelial cells, perycites, ganglion cells, lipid deposits, scar tissues, exudates, and thrombi. The average image obtained with a non-rigid registration of a stack of images could be properly segmented to enable the quantification of geometrical characteristics such as thickness, diameter, volume, or density that have diagnostic value in many retinal diseases.

## Data Availability

Not applicable.

## Supplementary Materials

The following supporting information can be downloaded at: https://www.mdpi.com/article/10.3390/diagnostics13132285/s1, Video S1: Image stack; Video S2: Nonrigid registration iterations; Video S3: Deformation field, rigid/nonrigid registration; Video S4: Deformation field, rigid/nonrigid registration.
